# Stochastic behaviors of an improved Gompertz tumor growth model with coupled two types noise

**DOI:** 10.1016/j.heliyon.2022.e11574

**Published:** 2022-11-11

**Authors:** Huijun Lv, Guitian He, Hui Cheng, Yun Peng

**Affiliations:** College of Mathematics and Physics, Center for Applied Mathematics of Guangxi Minzu University, Nanning 530006, China

**Keywords:** Improved Gompertz tumor growth model, Fokker-Planck equation, Colored noise, Skewness

## Abstract

In this work, an improved Gompertz tumor growth model has been introduced. The expressions of steady probability distributions (SPD) of stochastic Gompertz tumor growth models are studied by using the technique of Fokker-Planck equation (FPE), and their dynamic behaviors are also further investigated. Moreover, the expressions for mean, variance, skewness, as well as the mean first-passage time (MFPT) also have been derived. And the influence of noise intensity, correlation coefficient, and noise correlation time of SPD are further analyzed. It is worthy noting that the colored noise intensity has an important impact on SPD. Furthermore, adjusting birth and death parameters also significantly impact SPD, MFPT, mean, variance as well as skewness.

## 1 Introduction and background

A malignant tumor has been taken into account as a major killer threatening human beings. Therefore, studying the growth law of a malignant tumor is helpful for human beings to understand its growth characteristics and find measures to control and eliminate it [1], [2], [3], [4]. Considering quantitatively studying the law of tumor growth. It is necessary to establish a mathematical model to meet the growth and reproduction of tumor cells. Over recent years, scholars have put forward various mathematical models of tumor growth. Thus, the logistic growth model [5], [6], [7], [8], power law growth model [9], [10], [11] as well as Gompertz growth model [12], [13], [14] have been well built to describe tumor growth system.

A fundamental representation of cell growth, and more specifically, the representation of tumor cells, is frequently the logistic growth model [7], [8]. Thus, the tumor cell growth system could be appropriately established by

(1) $$\frac{dx}{\;dt}=ax-bx^{2},$$

in which *a* denotes the growth rate, *b* stands for the decay rate, and *x* indicates the tumor cell number.

In fact, owing to physiological limitations and the interaction between cells, the logistic model is sometimes inconsistent with the actual situation. To model naturally, a Gompertz growth model [12] with logarithmic functions can be considered as follows

(2) $$\frac{dx}{\;dt}=kx\ln\left(\frac{x}{N_{m}}\right),$$

in which *k* denotes the relative growth rate of tumor cells, $N_{m}$ represents environmental carrying capacity.

The logistic model (1) and Gompertz model (2) have been deeply discussed and studied in many previous pieces of literature. In recent years, Ref. [15] studied a solid tumor growth model described by the deterministic improved Gompertz model (3), which appears to be especially in line with the data on tumor growth. Its differential form is as follows

(3) $$\frac{dx}{\;dt}=\alpha x-\beta x\ln x,\;\;x(0)=x_{0},$$

where *α* and *β* respectively indicate the birth rate and death rate (α>0, β>0). *x* represents the tumor cell number which is a function of time *t*. And −βxln⁡x describes the cell death effect with the death rate *β*. Thus, for the aspect of modeling naturally, we will focus on the improved Gompertz growth model in this work. Nonexistence of migration and contact with other species, it uses an S-shaped function to describe the expansion of a population made up of a collection of individuals from one or more similar species.

Mathematical models of tumor growth have significantly developed over the past few decades. Among the proposed models, the model based on the deterministic case of ordinary differential equations has been widely used [5], [6], [7], [8], [9], [10], [11]. However, the growth of tumor cells in organisms requires consideration of variability between individuals and the randomness of the environment, which is inherent in any growth process [16]. In addition, there are some outside factors like temperature, radiotherapy, medications, etc. These factors have the potential to directly influence both the number of tumor cells and the rate of tumor growth [5]. But the deterministic model ignores the source of these variability factors. In order to take this environmental fluctuation and the differences between individuals into account, Ricciardi et al. proposed growing in a random environment so that the growth of tumors could be characterized by stochastic differential equations [17], [18], [19], [20], [21], [22]. These models include one or more noise terms, and the solutions of models are actually diffusion processes [16]. Accordingly, compared to the standard deterministic model, the stochastic improved Gompertz tumor growth model is more accurate in describing the actual situation of tumor cell growth.

Physically, it makes sense and is acceptable and straightforward that the effects of these outside elements are modeled by both additive and multiplicative noise, taking into account the stochastic characteristics of variations of these external factors. In general, the environment in which tumor cells grow, such as oxygen temperature and nutrients, is often affected by additive noise [23]. While some therapies, such as chemotherapy, surgery, and radiotherapy, are usually described by multiplicative noise [23]. Thus, there has been great much research on the effect of additive noise or multiplicative noise on tumor growth system [24], [25], [26], [27], [28], [29]. Ref. [24] studied the bacterium growth described by a logistic growth model with Gaussian colored noise. Ref. [25] investigated the SPD and MFPT of tumor growth systems under the influence of colored noise. While Ref. [27] studied a stochastic tumor cell growth system's MFPT and SPD under the impact of corrected colored noise. Recently, stochastic resonance phenomena induced by tumor growth systems under the impact of positional and environmental variables were studied in Ref. [28]. Tumor-immune responses to treatment have been investigated in Ref. [29] using deterministic and stochastic differential equation models. In addition, Albano et al. [15] investigated a solid tumor growth model with Gompertz's deterministic law and obtained some positive numerical findings for the particular situation of parathyroid tumors. Ref. [30] applied experimental data implanted into patient-derived uveal melanoma (patient-derived xenograft) mice to the improved Gompertz growth model in a randomized environment. Ref. [31] uses the experimental research data of breast cancer xenotransplantation, that is, the experimental data of BC297MONp5 tumor growth observed in the experimental group of mice, to verify the model by numerical simulation.

Motivated by the above research, it is worthwhile to investigate the stochastic dynamic behavior of an improved Gompertz tumor growth system with a coupling of Gaussian white noise and a colored noise. The structure of the rest of this work is organized as follows. In Section 2, an improved deterministic equation is briefly introduced to add the random environment to the Gompertz model, the Langevin equation corresponding to adding the noise term in the Gompertz theoretical model is introduced, and the approximate analytical expression of SPD will be derived. In Section 3, using numerical methods, the influences of system parameters, noise intensity, cross-correlation coefficient, and autocorrelation time of SPD, mean value, normalized variance, and normalized skewness will be extensively addressed and discussed. Finally, some main results and conclusions will be drawn in Section 4.

## 2 Deterministic and stochastic tumor growth model

It is well known that Eq. (3) describes a deterministic model without any fluctuations. In addition, one knows that the potential function plays a decisive role in molecular dynamics [32], [33], [34], [35], [36], [37]. And the monostable asymmetric situation deterministic potential function of the model (3) is written as

(4) $$V(x)=-\frac{2\alpha +\beta }{4}x^{2}+\frac{\beta }{2}x^{2}\ln x,$$

here V(x) has one minimum $x_{s}$. $x_{s}$ represents a diseased state, where the cell number is at a stable level. As a matter of convenience, take an unstable point $x_{u}$ of the deterministic system (3).

**Figure 1:**
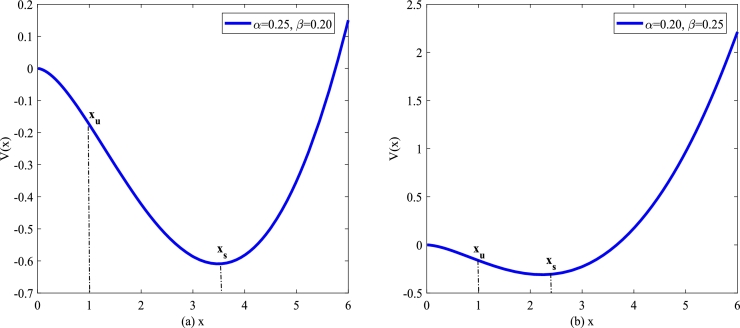
The potential function *V*(*x*) described by Eq. (4) for various *α* and *β*. (a) *α* = 0.25,*β* = 0.2; (b) *α* = 0.2,*β* = 0.25.

In Fig. 1(a) and Fig. 1(b), one can find that the equilibrium points of Eq. (4) strongly depend on *α* and *β*. Obviously, the depth and width of the potential well are influenced by system parameters *α* and *β*. The equilibrium points of Eq. (3) means the positions where the nonlinear force equals 0,

$$F(x)=V^{\prime }(x)=\alpha x-\beta x\ln x=0.$$

In fact, Eq. (3) is one of the most basic models to characterize the growth of tumor cells, which is an ideal model, assuming that tumor cells grow undisturbed. According to the previous analysis, in this section, we will focus on the stochastic dynamical characters of the improved Gompertz growth model with correlated Gaussian white noise and Gaussian colored noise. More precisely, we initially analyze the characteristics of the improved Gompertz growth model. In addition, taking into account the fact that the biological system is extremely complex and different interconnected as a whole series, studying the dynamic phenomena of a complex system can not ignore the correlation between noise. The correlation between multiplicative and additive noises is therefore assumed in this work (that is, they have a common origin).

### 2.1 Tumor growth model with cross-correlation Gaussian white noises

According to the previous discussion, in this subsection, we will focus on the tumor growth model with relevant both additive and multiplicative Gaussian white noise. Thus, from the perspective of statistical physics of the correlated noise system, the Langevin equation (LE) of the tumor growth system could be written as

(5) $$\left\{ \begin{array}{c} \frac{dx}{\;dt}=\alpha x-\beta x\ln x+x\varepsilon (t)+\Lambda (t),\\ x\left(t_{0}\right)=x_{0}. \end{array}\right.$$

In Eq. (5), we assume ε(t) and Λ(t) are zero mean Gaussian white noises with correlation function which meets [38] following properties

(6) $$\langle \varepsilon (t)\varepsilon (s)\rangle =2D_{1}\delta (t-s),\langle \Lambda (t)\Lambda (s)\rangle =2Q\delta (t-s),\langle \varepsilon (t)\Lambda (s)\rangle =\langle \Lambda (t)\varepsilon (s)\rangle =2\lambda_{1}\sqrt{D_{1}Q}\delta (t-s),$$

in which $D_{1}$ and *Q* stand for the noise intensities of multiplicative Gaussian white noise ε(t) and additive Gaussian white noise Λ(t), respectively. $\lambda_{1}$ ($0\leq |\lambda_{1}|\leq 1$) indicates the coupling coefficient between ε(t) and Λ(t).

Considering the fact that the cell number x≥0, the Fokker-Planck equation (FPE) [39], [40] corresponding to model (5) could be derived

(7) $$\frac{\partial P(x,t)}{\partial t}=-\frac{\partial [A(x)P(x,t)]}{\partial x}+\frac{\partial^{2}[B(x)P(x,t)]}{\partial x^{2}},$$

in which P(x,t) represents the probability density of the stochastic process defined by Eq. (5). And A(x) and B(x) in Eq. (7) denote drift coefficient and diffusion coefficient, respectively, defined by

(8) $$\left\{ \begin{array}{c} A(x)=\alpha x-\beta x\ln x+D_{1}x+\lambda_{1}\sqrt{D_{1}Q},\\ B(x)=D_{1}x^{2}+2\lambda_{1}\sqrt{D_{1}Q}x+Q. \end{array}\right.$$

Let $\frac{\partial P(x,t)}{\partial t}=0$, integral to the right side of Eq. (7). By using the technique reported by Refs. [40], [41], [42], [43], SPD corresponding to Eq. (5) could be derived as

(9) $$P_{st}(x)=\frac{N}{\sqrt{B(x)}}\exp\left\{\int \frac{A(x)}{B(x)}dx\right\}=\frac{N}{\sqrt{B(x)}}\exp\left[-\Phi_{1}(x)\right],$$

where *N* denotes a normalization constant, which satisfies the condition, $\int_{0}^{+\infty }P_{st}(x)dx=1.$

Using the expressions of A(x) and B(x) given by Eq. (8), one can derive the expression of SPD,

(10) $$P_{st}(x)=\left\{ \begin{array}{c} N{\left(D_{1}x^{2}+2\lambda_{1}\sqrt{D_{1}Q}x+Q\right)}^{c_{1}-\frac{1}{2}}\exp\left[f_{1}(x)+\frac{E_{1}}{\sqrt{\left(1-\lambda_{1}^{2}\right)D_{1}Q}}\arctan\left(\frac{D_{1}x+\lambda_{1}\sqrt{D_{1}Q}}{\sqrt{\left(1-\lambda_{1}^{2}\right)D_{1}Q}}\right)\right],\;0\leq \left|\lambda_{1}\right|<1,\\ \overset{˜}{N}{\left(\sqrt{D_{1}}x+\sqrt{Q}\right)}^{{\overset{˜}{c}}_{1}-1}\exp\left[{\overset{˜}{f}}_{1}(x)+\frac{{\overset{˜}{E}}_{1}}{\sqrt{D_{1}}x+\sqrt{Q}}\right],\;\left|\lambda_{1}\right|=1, \end{array}\right.$$

in which *N* and $\overset{˜}{N}$ denote two normalization constants, and some coefficients are given by

(11) $$\left\{ \begin{array}{c} f_{1}(x)=\frac{\beta }{4D_{1}}x^{2}-\left(\frac{2\beta }{D_{1}}+\frac{\lambda_{1}\beta \sqrt{D_{1}Q}}{D_{1}^{2}}\right)x,\\ C_{1}=\frac{\alpha }{2D_{1}}+\frac{3\beta }{4D_{1}}+\frac{2\lambda_{1}\beta \sqrt{D_{1}Q}}{D_{1}^{2}}+\frac{\left(4\lambda_{1}^{2}-1\right)\beta Q}{4D_{1}^{2}}+\frac{1}{2},\\ E_{1}=\frac{\left(2-4\lambda_{1}^{2}\right)\beta Q}{D_{1}}-\frac{(2\alpha +3\beta )\lambda_{1}\sqrt{D_{1}Q}}{2D_{1}}+\frac{\left(3-4\lambda_{1}^{2}\right)\lambda_{1}\beta Q\sqrt{D_{1}Q}}{2D_{1}^{2}},\\ {\overset{˜}{f}}_{1}(x)=\frac{\beta }{4D_{1}}x^{2}-\left(\frac{2\beta }{D_{1}}+\frac{\beta \sqrt{D_{1}Q}}{{D_{1}}^{2}}\right)x-\frac{\beta Q+2\beta \sqrt{D_{1}Q}}{D_{1}^{2}},\\ {\overset{˜}{C}}_{1}=\frac{\alpha }{D_{1}}+\frac{3\beta }{2D_{1}}+\frac{\beta Q}{2{D_{1}}^{2}}+\frac{4\beta \sqrt{D_{1}Q}}{{D_{1}}^{2}}+1,\\ {\overset{˜}{E}}_{1}=\frac{\alpha \sqrt{Q}}{D_{1}}+\frac{3\beta \sqrt{Q}}{2D_{1}}+\frac{2\beta Q\sqrt{D_{1}}}{D_{1}^{2}}+\frac{\beta Q\sqrt{Q}}{2{D_{1}}^{2}}. \end{array}\right.$$

The exponential part of Eq. (9) corresponds to the modified potential function, denoted as $\Phi_{1}$. The generalized potential function [44] could be modified as

(12) $$\Phi_{1}(x)=-\int \frac{A\left(x\right)}{B\left(x\right)}dx.$$

After algebra calculation with Eqs. (8), (12), the expression of the modified potential function could be obtained as

(13) $$\Phi_{1}(x)=\left\{ \begin{array}{c} -f_{1}(x)-C_{1}\ln\left(D_{1}x^{2}+2\lambda_{1}\sqrt{D_{1}Q}x+Q\right)-\frac{E_{1}}{\sqrt{\left(1-\lambda_{1}^{2}\right)D_{1}Q}}\arctan\frac{D_{1}x+\lambda_{1}\sqrt{D_{1}Q}}{\sqrt{\left(1-\lambda_{1}^{2}\right)D_{1}Q}},0\leq \left|\lambda_{1}\right|<1,\\ -{\overset{˜}{f}}_{1}(x)-{\overset{˜}{C}}_{1}\ln\left(\sqrt{D_{1}}x+\sqrt{Q}\right)-\frac{{\overset{˜}{E}}_{1}}{\sqrt{D_{1}}x+\sqrt{Q}},\;\left|\lambda_{1}\right|=1, \end{array}\right.$$

where $C_{1}$, ${\overset{˜}{C}}_{1}$, $E_{1}$, ${\overset{˜}{E}}_{1}$, $f_{1}(x)$, and ${\overset{˜}{f}}_{1}(x)$ in Eq. (13) are given by Eq. (11).

**Figure 2:**
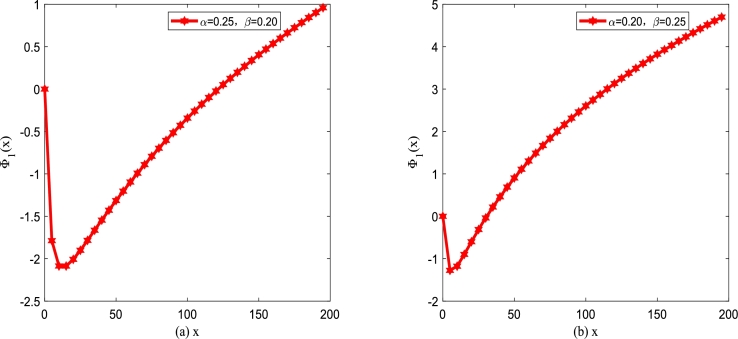
The modified potential function Φ_1_(*x*) described by Eq. (13) for different *α* and *β* with *D*_1_ = 0.25,*Q* = 0.15, and *λ*_1_ = 0.1. (a) *α* = 0.25,*β* = 0.2; (b) *α* = 0.2,*β* = 0.25.

It is well known that the interaction between the immune system and tumor cells is a typical competitive behavior in molecular biology [45]. In addition, internal and external random disturbances of the biological system affect the control of the immune system tumor cells. Tumor cells are required to overcome the corresponding constraints if tumor cells want to escape the surveillance of the immune system. This phenomenon is a consequence of physics as the motion of particles in a potential well. Therefore, the growth process of tumor cell number with particles moving in modified potential $\Phi_{1}(x)$ in Eq. (13) is simulated, as shown in Figs. 2(a) and (b).

**Figure 3:**
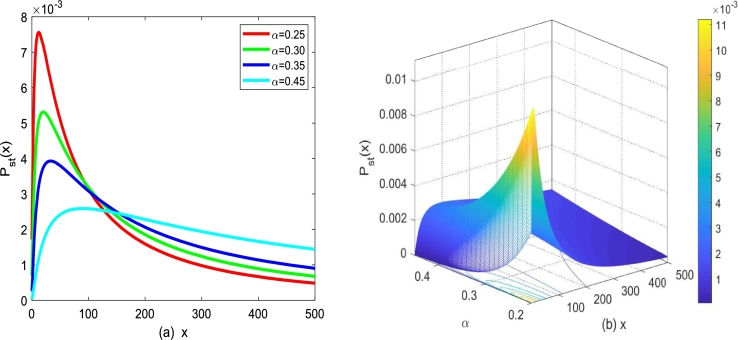
(a) *P*_*st*_(*x*) described by Eq. (9) for different *α* with *β* = 0.1,*D*_1_ = 0.25,*Q* = 0.15,*λ*_1_ = 0.2, (b) three-dimensional function *P*_*st*_(*x*) described by Eq. (9) versus *α* and *x*.

Based on Eq. (9), for the asymmetric monostable situation model, the influence of correlation Gaussian noise on stationary probability density can be analyzed. Moreover, the influence of cross-correlation coefficient $\lambda_{1}$, born rate *α*, death rate *β* as well as noise parameters on $P_{st}(x)$ are depicted in Figure 3, Figure 6.

Fig. 3 plots the effect of the birth rate *α* of SPD under correlated additive Λ(t) and multiplicative Gaussian white noise ε(t). For $\beta =0.2,D_{1}=0.25,Q=0.15$ and $\lambda_{1}=0.1$, Fig. 3(a) plots the graph of $P_{st}(x)-x$ with different *α*. $P_{st}(x)$ starts to increase and then decreases with *x* increasing depicted in Fig. 3(a). In addition, with birth rate *α* increasing, the maximum value of SPD declines, and its position of maximum value of SPD gradually shifts to the right. Fig. 3(b) presents a three-dimensional diagram of the functional surface of $P_{st}(x)$, a function of number of tumor cells *x* and birth rate *α*. Significantly, results depicted in Fig. 3(b) are consistent with the analysis in Fig. 3(a).

In conclusion, one can find that the size of birth rate *α* greatly affects the stability of tumor cell growth (*x* represents the number of tumor cells). If the death rate *β* is known or can be estimated beforehand, to indicate the effect of treatment, we can seek time-dependent functions that influence the birth rate. In particular, since *α* represents the birth rate, the addition of an additional term modifies birth rate *α* by taking into account the impact on cell proliferation. Birth rates increase when the additional term has a negative value, while birth rates fall when the additional term has a positive value. Moreover, only if the value of the additional term is greater than the birth rate *α*, the number of tumor cells will be reduced, to obtain a better therapeutic effect.

**Figure 4:**
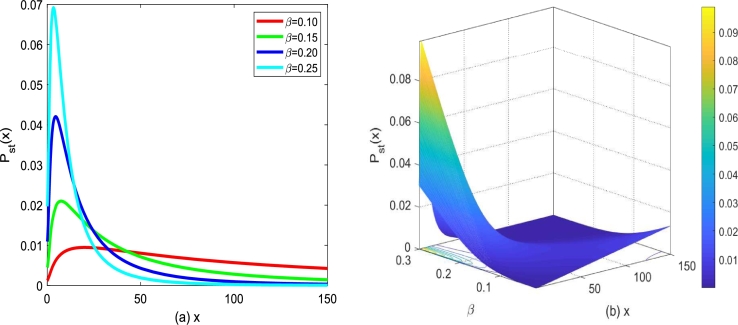
(a) *P*_*st*_(*x*) described by Eq. (9) for different *β* with *α* = 0.3,*D*_1_ = 0.25,*Q* = 0.15,*λ*_1_ = 0.2, (b) *P*_*st*_(*x*) described by Eq. (9) versus *β* and *x*.

Fig. 4 shows the influence of the death rate *β* on SPD under ε(t) and Λ(t) with properties (6). For $\alpha =0.3,D_{1}=0.25,Q=0.15$ and $\lambda_{1}=0.1$, Fig. 4(a) plots the graph of $P_{st}(x)-x$ for different *β*. From Fig. 4(a), one also can find that $P_{st}(x)$ starts to increase and then decreases with *x* increasing, and the curvilinear shape changes if *β* changes. With the death rate *β* increasing, the maximum value of SPD increases, and its position of maximum value of SPD gradually shifts to the left. Fig. 4(b) presents a three-dimensional diagram of the functional surface of $P_{st}(x)$ versus *x* and *β*, and the results obtained are consistent with the analysis in Fig. 4(a).

In conclusion, one can find that the change of the death parameter *β* also greatly affects the stability of tumor cell growth. We can seek time-dependent functions that influence the death rate *β* to indicate the effect of therapy if the birth rate *α* is known or can be anticipated in advance. In particular, because *β* represents the death rate, adding an additional term affects the death rate *β* by including the cell death impact. A decrease in the death rate is indicated by a negative value of the additional term, whereas an increase in the death rate is shown by a positive value of the additional term. Additionally, it is best to view the additive term as having a positive value to have a better therapeutic impact.

**Figure 5:**
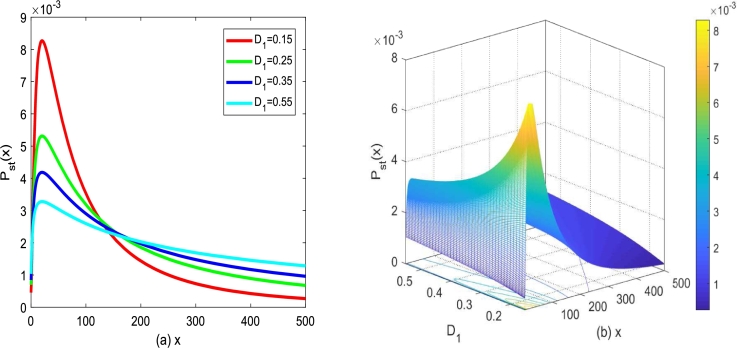
(a) *P*_*st*_(*x*) described by Eq. (9) for different *D*_1_ with *α* = 0.3,*β* = 0.1,*Q* = 0.15,*λ*_1_ = 0.2, (b) *P*_*st*_(*x*) described by Eq. (9) versus *D*_1_ and *x*.

Fig. 5 plots the influence of the multiplicative noise intensity $D_{1}$ on SPD under ε(t) and Λ(t) with properties (6). For $\alpha =0.3,D_{1}=0.25,Q=0.15$ and $\lambda_{1}=0.1$, Fig. 5(a) depicts the graph of $P_{st}(x)-x$ for different multiplicative noise intensity $D_{1}$. One can find that SPD, $P_{st}(x)$, presents a unimodal phenomenon, which means that $P_{st}(x)$ starts to increase and then decreases with *x* increasing. However, it is found that the extreme value of $P_{st}(x)$ decreases with the increase of $D_{1}$. In addition, Fig. 5(b) presents the three-dimensional graph of the function surface of $P_{st}(x)$ versus the number of tumor cells *x* and multiplicative noise intensity $D_{1}$, whose showing results is consistent with the analysis results in Fig. 5(a). Since x→0 represents a decrease in the number of tumor cells, from this perspective, the change of the intensity of multiplicative noise will result in the change of the number of tumor cells. Meanwhile, multiplicative noise is external noise that can be controlled artificially. For example, in real life, the intensity of multiplicative noise can be controlled by increasing the dose of anticancer drugs, prolonging the action time of drugs and increasing the intensity of radiation in radiotherapy.

**Figure 6:**
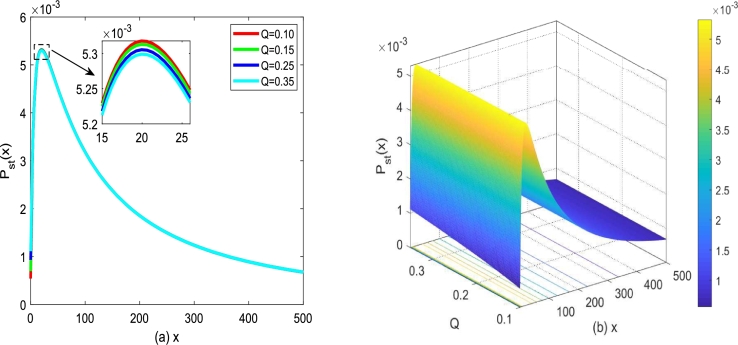
(a) *P*_*st*_(*x*) described by Eq. (9) for different *Q* with *α* = 0.3,*β* = 0.1,*D*_1_ = 0.25,*λ*_1_ = 0.2, (b) *P*_*st*_(*x*) described by Eq. (9) versus *Q* and *x*.

Fig. 6 plots the effect of additive noise intensity *Q* variation on SPD under ε(t) and Λ(t) with properties (6). As shown in Fig. 6 (a), $P_{st}(x)$ starts to increases greatly, and then decreases gradually with the increase of *x* for noise intensity *Q* fixed. Significantly, with the increase of additive noise intensity *Q*, the shape of SPD curve, presenting an asymmetric single-peak structure, almost does not change depicted in Fig. 6(a). However, the maximum value of $P_{st}(x)$ decreases slightly with *Q* increasing. In addition, Fig. 6(b) presents the three-dimensional graph of function surface where $P_{st}(x)$ varies with the number of tumor cells *x* and additive noise intensity *Q*, and the results obtained are consistent with those analyzed in Fig. 6(a). Additive noise is a kind of internal noise, which is generated in the system. Therefore, from this aspect, the change of additive noise intensity has little influence on the change of tumor cell number. In the process of tumor cell growth, if the tumor cell survival environment changes, the intensity *Q* of additive noise will also change. Precisely, if patients could keep relatively physical health and a hopeful mood, the intensity of additive noise can be controlled by altering the patient's immunity.

### 2.2 Tumor growth model under cross-correlation colored and white noises excitation

In actual circumstances, most noises in nature are colored, and their auto-correlation time is not zero. In this subsection, we consider multiplicative noise to be Gaussian colored noise in the improved Gompertz tumor growth system. Thus, the Langevin equation (LE) of the tumor growth system under cross-correlation colored and white noise excitation could be written as

(14) $$\left\{ \begin{array}{c} \frac{dx}{\;dt}=\alpha x-\beta x\ln x+x\xi (t)+\eta (t),\\ x\left(t_{0}\right)=x_{0}, \end{array}\right.$$

in which ξ(t) and η(t) are zero-mean Gaussian noises with correlation function [46], [47], [48] written as

(15) $$\langle \xi (t)\xi (s)\rangle =\frac{D_{2}}{\tau }\exp\left(-\frac{\left|t-s\right|}{\tau }\right),\langle \eta (t)\eta (s)\rangle =2Q\delta (t-s),\langle \xi (t)\eta (s)\rangle =\langle \eta (t)\xi (s)\rangle =2\lambda_{2}\sqrt{D_{2}Q}\delta (t-s),$$

where $\lambda_{2}$ represents the degree of correlation between the noise ξ(t) and η(t). $D_{2}$ and *Q* denote the intensities of the noise ξ(t) and η(t), respectively. Using the statistical properties of Eq. (15), thus, Eq. (14) could be equivalent to the following two-dimensional Markov process

$$\left\{ \begin{array}{c} \frac{dx}{\;dt}=\alpha x-\beta x\ln x+x\xi (t)+\eta (t),\\ \frac{d\xi }{dt}=-\frac{1}{\tau }\xi (t)+\frac{1}{\tau }\varsigma (t), \end{array}\right.$$

in which ς(t) denotes another Gaussian white noise meeting following statistical condition,

$$\langle \varsigma (t)\rangle =0,\langle \varsigma (t)\varsigma (s)\rangle =2D_{2}\delta (t-s).$$

Using Novikov's theorem and together with unified colored noise approximation theory, FPE corresponding to Eq. (14) could be derived as

(16) $$\frac{\partial P(x,t)}{\partial t}=-\frac{\partial [G(x)P(x,t)]}{\partial x}+\frac{\partial^{2}[H(x)P(x,t)]}{\partial x^{2}},$$

in which G(x) and H(x) denote drift coefficient and diffusion coefficient, respectively, defined as

(17) $$\left\{ \begin{array}{c} G(x)=\frac{\alpha x-\beta x\ln x}{1+\tau \beta }+\frac{D_{2}x+\lambda_{2}\sqrt{D_{2}Q}}{{\left(1+\tau \beta \right)}^{2}},\\ H(x)=\frac{D_{2}x^{2}+2\lambda_{2}\sqrt{D_{2}Q}x+Q}{{\left(1+\tau \beta \right)}^{2}}. \end{array}\right.$$

Using the technique in Ref. [43], the SPD of Eq. (16) could be written as

(18) $$P_{st}(x)=\frac{M}{\sqrt{H(x)}}\exp\left\{\int \frac{G(x)}{H(x)}\mathrm{dx}\right\}=\frac{M}{\sqrt{H(x)}}\exp\left[-\Phi_{2}(x)\right],$$

where *M* is a normalization constant, which meets the condition given by

$$\int_{0}^{+\infty }P_{st}(x)dx=1.$$

After some calculation, one can obtain SPD $P_{st}(x)$ given by

(19) $$P_{st}(x)=\left\{ \begin{array}{c} M(1+\tau \beta ){\left(D_{2}x^{2}+2\lambda_{2}\sqrt{D_{2}Q}x+Q\right)}^{C_{2}-\frac{1}{2}}\times \exp\left\{f_{2}(x)+\frac{E_{2}}{\sqrt{\left(1-{\lambda_{2}}^{2}\right)DQ}}\arctan\frac{D_{2}x+\lambda_{2}\sqrt{D_{2}Q}}{\sqrt{\left(1-{\lambda_{2}}^{2}\right)D_{2}Q}}\right\},\;0\leq \left|\lambda_{2}\right|<1\\ \overset{˜}{M}(1+\tau \beta ){\left(\sqrt{D_{2}}x+\sqrt{Q}\right)}^{{\overset{˜}{C}}_{2}-1}\times \exp\left\{{\overset{˜}{f}}_{2}(x)+\frac{{\overset{˜}{E}}_{2}}{\sqrt{D_{2}}x+\sqrt{Q}}\right\},\;\left|\lambda_{2}\right|=1 \end{array}\right.$$

where *M*, $\overset{˜}{M}$ are two normalization constants, and some coefficients are given by

(20) $$\left\{ \begin{array}{c} f_{2}(x)=\frac{\beta \left(1+\tau \beta \right)}{4D_{2}}x^{2}-\left[\frac{2\beta \left(1+\tau \beta \right)}{D_{2}}+\frac{\lambda_{2}\beta \sqrt{D_{2}Q}\left(1+\tau \beta \right)}{{D_{2}}^{2}}\right]x,\\ C_{2}=\frac{\alpha \left(1+\tau \beta \right)}{2D_{2}}+\frac{3\beta \left(1+\tau \beta \right)}{4D_{2}}+\frac{2\lambda_{2}\beta \sqrt{D_{2}Q}\left(1+\tau \beta \right)}{{D_{2}}^{2}}+\frac{\beta Q\left(1+\tau \beta \right)(4{\lambda_{2}}^{2}-1)}{4{D_{2}}^{2}}+\frac{1}{2},\\ E_{2}=\frac{\beta Q\left(1+\tau \beta \right)\left(2-4{\lambda_{2}}^{2}\right)}{D_{2}}-\frac{\lambda_{2}\sqrt{D_{2}Q}\left(1+\tau \beta \right)(2\alpha +3\beta )}{2D_{2}}+\frac{\lambda_{2}\beta Q\sqrt{D_{2}Q}\left(1+\tau \beta \right)(3-4{\lambda_{2}}^{2})}{2{D_{2}}^{2}},\\ {\overset{˜}{f}}_{2}(x)=\frac{\beta \left(1+\tau \beta \right)}{4D_{2}}x^{2}-\left[\frac{2\beta \left(1+\tau \beta \right)}{D_{2}}+\frac{\beta \sqrt{D_{2}Q}\left(1+\tau \beta \right)}{{D_{2}}^{2}}\right]x-\frac{\beta \left(Q+2\sqrt{D_{2}Q}\right)\left(1+\tau \beta \right)}{{D_{2}}^{2}},\\ {\overset{˜}{C}}_{2}=\frac{\alpha \left(1+\tau \beta \right)}{D_{2}}+\frac{3\beta \left(1+\tau \beta \right)}{2D_{2}}+\frac{\beta Q\left(1+\tau \beta \right)}{2{D_{2}}^{2}}+\frac{4\beta \sqrt{D_{2}Q}\left(1+\tau \beta \right)}{{D_{2}}^{2}}+1,\\ {\overset{˜}{E}}_{2}=\frac{\alpha \sqrt{Q}\left(1+\tau \beta \right)}{D_{2}}+\frac{3\beta \sqrt{Q}\left(1+\tau \beta \right)}{2D_{2}}+\frac{2\beta Q\sqrt{D_{2}}\left(1+\tau \beta \right)}{{D_{2}}^{2}}+\frac{\beta Q\sqrt{Q}\left(1+\tau \beta \right)}{2{D_{2}}^{2}}. \end{array}\right.$$

The exponential part of Eq. (18) corresponds to the modified potential function [44] of the system, denoted as $\Phi_{2}(x)$ given by

(21) $$\Phi_{2}(x)=-\int \frac{G\left(x\right)}{H\left(x\right)}dx.$$

Using Eq. (17) and Eq. (21), the expression of the modified potential function can be obtained as

(22) $$\Phi_{2}(x)=\left\{ \begin{array}{c} -f_{2}(x)-C_{2}\ln\left(D_{2}x^{2}+2\lambda_{2}\sqrt{D_{2}Q}x+Q\right)-\frac{E_{2}}{\sqrt{\left(1-{\lambda_{2}}^{2}\right)D_{2}Q}}\arctan\frac{D_{2}x+\lambda_{2}\sqrt{D_{2}Q}}{\sqrt{\left(1-{\lambda_{2}}^{2}\right)D_{2}Q}},\;0\leq \left|\lambda_{2}\right|<1\\ -{\overset{˜}{f}}_{2}(x)-{\overset{˜}{C}}_{2}\ln\left(\sqrt{D_{2}}x+\sqrt{Q}\right)-\frac{{\overset{˜}{E}}_{2}}{\sqrt{D_{2}x+\sqrt{Q}}},\;\left|\lambda_{2}\right|=1 \end{array}\right.$$

where $C_{2}$, ${\overset{˜}{C}}_{2}$, $E_{2}$, ${\overset{˜}{E}}_{2}$, $f_{2}(x)$, and ${\overset{˜}{f}}_{2}(x)$ in Eq. (22) are given by Eq. (20).

**Figure 7:**
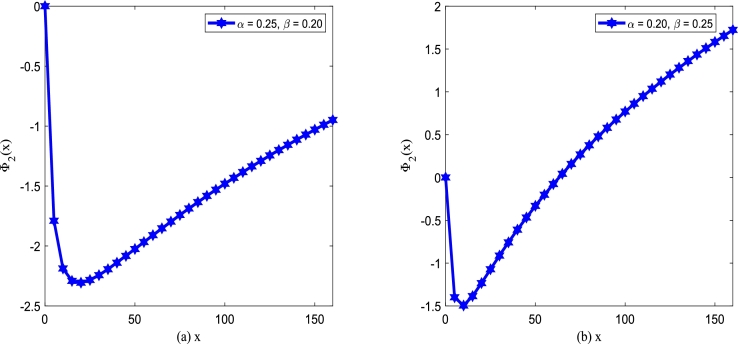
The modified potential function Φ_2_(*x*) described by Eq. (22) for different *α* and *β* with *D*_2_ = 0.35,*Q* = 0.15,*λ*_2_ = 0.1 and *τ* = 0.2. (a) *α* = 0.25,*β* = 0.2; (b) *α* = 0.2,*β* = 0.25.

Therefore, under the joint drive of Gaussian white noise and colored noise, the growth process of tumor cell number of particles moving in modified potential $\Phi_{2}(x)$ is simulated, as depicted in Fig. 7(a) and Fig. 7(b). The modified potential function $\Phi_{2}(x)$ is a single well affected by birth rate *α* and death rate *β* depicted in Fig. 7.

According to Eq. (16), one can also obtain Kolmogorov equation given by

(23) $$\frac{\partial P\left(x,t\right)}{\partial t}=-\frac{\partial \left[G\left(x\right)P\left(x,t\right)\right]}{\partial x}+\frac{\partial^{2}\left[H\left(x\right)P\left(x,t\right)\right]}{\partial x^{2}}.$$

For the stationary case, the FPE meets the condition given by $\frac{\partial P\left(x,t\right)}{\partial t}=0$.

From Eq. (23), one can also derive SPD $P_{st}(x)$,

(24) $$\frac{\partial \left[G\left(x\right)P_{st}\left(x\right)\right]}{\partial x}-\frac{\partial^{2}\left[H\left(x\right)P_{st}\left(x\right)\right]}{\partial x^{2}}=0.$$

Due to the complexity of the solution of SPD $P_{st}(x)$, the extreme value of SPD $P_{st}(x)$ at extreme point, $x=x_{m}$, will be addressed first. Due to the fact that it satisfies $\frac{dP_{st}\left(x_{m}\right)}{dt}=0$ and $P_{st}\left(x_{m}\right)\neq 0$, using Eq. (24), one has

$$0=-G\left(x_{m}\right)P_{st}\left(x_{m}\right)+\frac{d\left[H\left(x_{m}\right)P_{st}\left(x_{m}\right)\right]}{dx}=\left[-G\left(x_{m}\right)+\frac{dH\left(x_{m}\right)}{dx}\right]P_{st}\left(x_{m}\right)+H\left(x_{m}\right)\frac{dP_{st}\left(x_{m}\right)}{dx}.$$

Thus, one can also obtain

(25) $$-G\left(x_{m}\right)+\frac{dH\left(x_{m}\right)}{dx}=0.$$

In short, Eq. (25) can be written as

$$G\left(x_{m}\right)-H^{\prime }\left(x_{m}\right)=0.$$

Therefore, one has

(26) $$\frac{\alpha x_{m}}{1+\tau \beta }+\frac{\lambda_{2}\sqrt{D_{2}Q}}{{\left(1+\tau \beta \right)}^{2}}-\frac{\beta x_{m}\ln x_{m}}{1+\tau \beta }-\frac{D_{2}x_{m}}{{\left(1+\tau \beta \right)}^{2}}=0.$$

**Figure 8:**
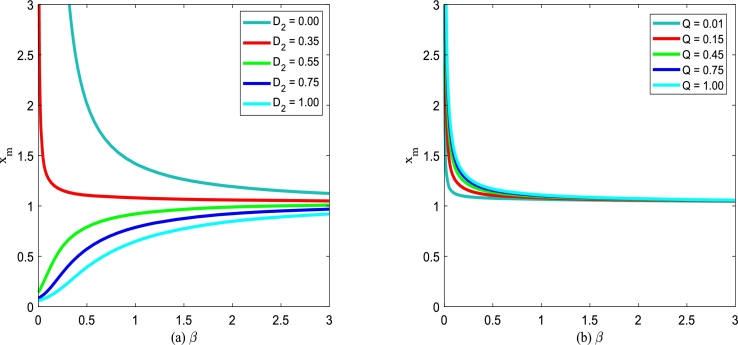
(a) Extreme point *x*_*m*_ of SPD versus the death rate *β* for various *D*_2_ with *Q* = 0.15,*λ*_2_ = 0.1 and *τ* = 0.2; (b) Extreme point *x*_*m*_ of SPD versus the death parameter *β* for various *Q* with *D*_2_ = 0.35,*λ*_2_ = 0.1 and *τ* = 0.2.

**Figure 9:**
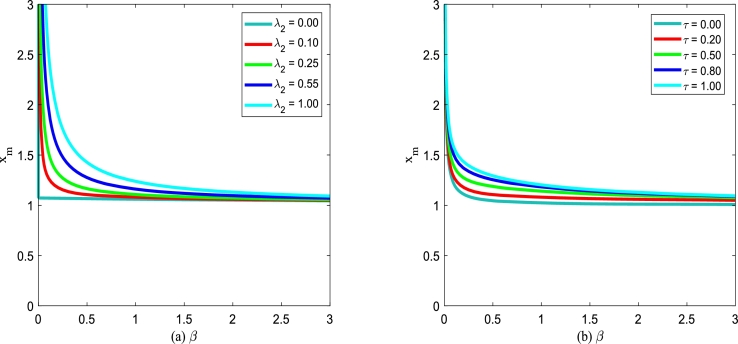
(a) Extreme point *x*_*m*_ of SPD versus the death rate *β* for various *λ* with *D*_2_ = 0.35,*Q* = 0.15 and *τ* = 0.2; (b) Extreme point *x*_*m*_ of SPD versus the death rate *β* for various *τ* with *D*_2_ = 0.35,*Q* = 0.15 and *λ* = 0.1.

Using Eq. (26), the steady state of the extreme value of SPD will be discussed. Due to the interference of noise, a stochastic system is distinct from a deterministic system. Fig. 8 and Fig. 9 show the extreme point $x_{m}$ of SPD of model (14) for various additive noise intensity *Q*, multiplicative noise intensity $D_{2}$, correlation time *τ* and cross-correlation intensity $\lambda_{2}$ between noises as a function of death rate *β*. For a convenient description of the extreme point $x_{m}$ versus the death rate *β*, the birth rate *α* is taken as 0.15.

In Fig. 8(a), parameters are taken as additive noise intensity Q=0.15, correlation coefficient $\lambda_{2}=0.1$ and correlation time τ=0.2, respectively. The extreme point $x_{m}$ decreases gradually, and then tends to be stable with the death rate *β* increasing for $D_{2}\leq 0.35$. While the extreme point $x_{m}$ increases gradually first and then tends to be stable as the death parameter *β* increases for $D_{2}>0.35$. Furthermore, $x_{m}$ decreases as noise intensity $D_{2}$ increases for fixed death rate *β*. In Fig. 8(b), parameters are taken as multiplicative noise intensity $D_{2}=0.35$, correlation coefficient $\lambda_{2}=0.1$ and correlation time τ=0.2, respectively. As shown in Fig. 8(b), the extreme point $x_{m}$ decreases gradually, and then tends to be stable with the death rate *β* increasing for fixed additive noise intensity *Q*. In addition, the extreme point $x_{m}$ increases as additive noise intensity *Q* increases.

In Fig. 9(a), the noise intensities are taken as $D_{2}$ = 0.35, *Q* = 0.15, and correlation time τ=0.2. The extreme point $x_{m}$ decreases first and then keeps stable with the death rate *β* increasing for the fixed correlation coefficient $\lambda_{2}$. With the change of the correlation coefficient $\lambda_{2}$, the shape of the $x_{m}$ with respect to *β* almost does not change. However, the extreme point $x_{m}$ increases gradually, as the correlation coefficient $\lambda_{2}$ goes from 0 to 1. Similarly, Fig. 9(b) also displays the analogous tendency for the noise intensities $D_{2}$= 0.35, *Q* = 0.15, and correlation coefficient $\lambda_{2}$= 0.1. The extreme point $x_{m}$ decreases first and then keeps stable with the death rate *β* increasing for the fixed correlation time *τ* depicted in Fig. 9(b). With the change of the correlation time *τ*, the shape of the $x_{m}$ with respect to *β* almost does not change. However, the extreme point $x_{m}$ at the same position becomes larger with the correlation time *τ* increasing.

Numerical simulation will be used to discuss the dynamics behavior of the improved Gompertz tumor growth system. Additionally, a deeper analysis of the impact of noise and system characteristics on SPD also will be conducted. The numerical simulation method of Gaussian noise is presented below. Initially, the following formula is used for the calculation to create two sets of random numbers $\omega =\{\omega_{i}\}$ and $\xi =\{\xi_{i}\}$ that follow the standard normal distribution [49],

(27) $$\left\{ \begin{array}{c} U_{i}=\sqrt{2D_{2}\Delta t}\omega_{i},\\ V_{i}=\sqrt{2Q\Delta t}\left(\lambda_{2}\omega_{i}+\sqrt{1-{\lambda_{2}}^{2}}\xi_{i}\right), \end{array}\right.$$

where $D_{2}$ and *Q* are noise intensity, Δ*t* is discrete time step. Furthermore, the numerical method of Eq. (14) resorts to the fourth order Runge-Kutta method [50], [51] given by

(28) $$\left\{ \begin{array}{c} x_{i+1}=x_{i}+\frac{\Delta t}{6}\left(h_{1}+2h_{2}+2h_{3}+h_{4}\right)+U_{i},\\ \xi_{i+1}=\xi_{i}+\frac{\Delta t}{6}\left(k_{1}+2k_{2}+2k_{3}+k_{4}\right)+\frac{1}{\tau }V_{i}, \end{array}\right.$$

here $h_{1}$, $h_{2}$, $h_{3}$, $h_{4}$, $k_{1}$, $k_{2}$, $k_{3}$ and $k_{4}$ are obtained by the expressions

(29) $$\left\{ \begin{array}{c} h_{1}=\alpha x_{i}-\beta x_{i}\ln x_{i}+x_{i}\xi_{i},\\ h_{2}=\alpha \left(x_{i}+\frac{\Delta t}{2}h_{1}+V_{i}\right)-\beta \left(x_{i}+\frac{\Delta t}{2}h_{1}+V_{i}\right)\ln\left(x_{i}+\frac{\Delta t}{2}h_{1}+V_{i}\right)+\left(x_{i}+\frac{\Delta t}{2}h_{1}+V_{i}\right)\left(\xi_{i}+\frac{\Delta t}{2}k_{1}+U_{i}\right),\\ h_{3}=\alpha \left(x_{i}+\frac{\Delta t}{2}h_{2}+V_{i}\right)-\beta \left(x_{i}+\frac{\Delta t}{2}h_{2}+V_{i}\right)\ln\left(x_{i}+\frac{\Delta t}{2}h_{2}+V_{i}\right)+\left(x_{i}+\frac{\Delta t}{2}h_{2}+V_{i}\right)\left(\xi_{i}+\frac{\Delta t}{2}k_{2}+U_{i}\right),\\ h_{4}=\alpha \left(x_{i}+\Delta th_{3}+V_{i}\right)-\beta \left(x_{i}+\Delta th_{3}+V_{i}\right)\ln\left(x_{i}+\Delta th_{3}+V_{i}\right)+\left(x_{i}+\Delta th_{3}+V_{i}\right)\left(\xi_{i}+\Delta th_{3}+U_{i}\right),\\ k_{1}=-\frac{1}{\tau }\xi_{i},\\ k_{2}=-\frac{1}{\tau }\left(\xi_{i}+\frac{\Delta t}{2}k_{1}+U_{i}\right),\\ k_{3}=-\frac{1}{\tau }\left(\xi_{i}+\frac{\Delta t}{2}k_{2}+U_{i}\right),\\ k_{4}=-\frac{1}{\tau }\left(\xi_{i}+\Delta tk_{3}+U_{i}\right). \end{array}\right.$$

**Figure 10:**
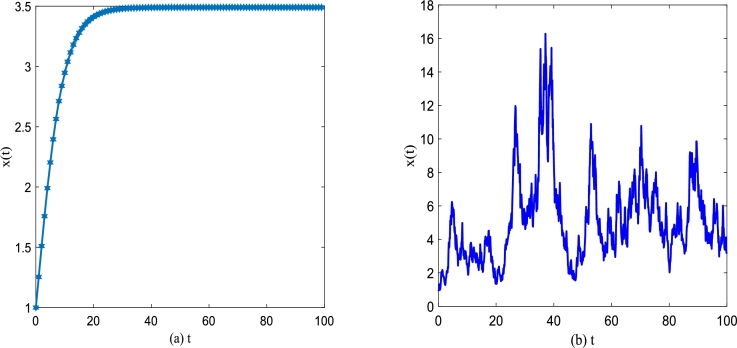
(a) No noise interferes with the discrete-time sequence diagram of Eq. (3) for *α* = 0.25,*β* = 0.2; (b) The discrete-time sequence diagram of Eq. (5) under gaussian white noise for *α* = 0.25,*β* = 0.2,*D*_1_ = 0.25,*Q* = 0.15, and *λ*_1_ = 0.1.

**Figure 11:**
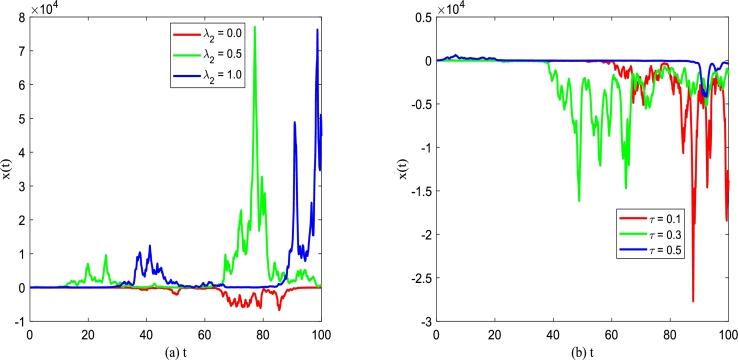
Discrete time series of improved Gompertz tumor growth system (14). (a) *α* = 0.25,*β* = 0.2,*D*_2_ = 0.35,*Q* = 0.15,*τ* = 0.2; (b) *α* = 0.25,*β* = 0.2,*D*_2_ = 0.35,*Q* = 0.15,*λ*_2_ = 0.1.

For investigation of the effect of white noise and colored noise on the unsteady state solution P(x,t), the evolution behavior of the system without noise disturbance and the evolution behavior of the system with Gaussian white noise disturbance are analyzed by simulation as shown in Fig. 10 and Fig. 11, respectively. For the sake of simulation, we assume $t_{0}=0,x_{0}=1$.

Fig. 10(a) and (b) depict the sequence diagram of the number of tumor cells *x* governed by Eq. (3) changing with time *t* without and with noise interference, respectively. One can find that Fig. 10(a) displays *x* is a monotone increasing function versus time *t* for t≈30 in absence of noise. Once *t* exceeds this value, *x* tends to a stable value if *t* keeps increasing, which also shows the existence of a stable solution consistently. From Fig. 10(b), one can also find that the addition of noise makes *x* fluctuate randomly around the original deterministic solution. It can be found that the existence of noise brings some additional characteristics to the improved Gompertz tumor growth system.

Fig. 11 demonstrates discrete time series of tumor growth system (14) in presence of both gaussian white noise and color noise. In Fig. 11, one can find that x(t) no longer changes regularly with *t*, but fluctuates randomly near the original deterministic solution. In addition, compared with discrete time series under just Gaussian white noise shown in Fig. 10(b), x(t) fluctuation with *t* also changes in a new way with two types of noise. From Fig. 11(a) and Fig. 11(b), x(t) fluctuates with time *t* presenting some new characteristics for the change of the correlation between two types of noise.

**Figure 12:**
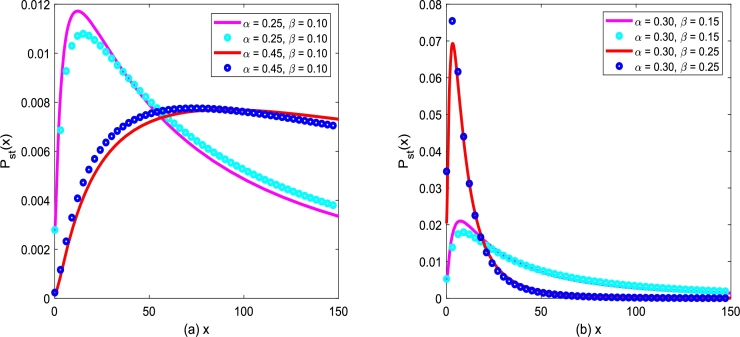
SPD, *P*_*st*_(*x*), governed by Eq. (5). The expression of SPD (9) is depicted by solid lines, while its numerical simulations are depicted by ∘. The parameters are taken as *λ*_1_ = 0.2,*D*_1_ = 0.25,*Q* = 0.15. (a) *α* = 0.25,*β* = 0.1 (a solid magenta line); *α* = 0.45,*β* = 0.1 (azure ∘); (b) *α* = 0.3,*β* = 0.15 (a solid red line); *α* = 0.3,*β* = 0.25 (blue ∘).

**Figure 13:**
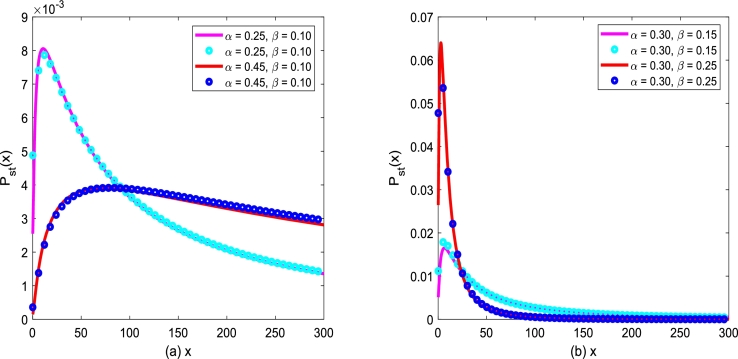
SPD, *P*_*st*_(*x*), governed by (14). The expression of SPD (18) is depicted by solid lines, while its numerical results are depicted by ∘. The parameters are taken as *λ*_2_ = 0.2,*τ* = 0.1,*D*_2_ = 0.35,*Q* = 0.15. (a) *α* = 0.25,*β* = 0.1 (a solid magenta line); *α* = 0.45,*β* = 0.1 (azure ∘); (b) *α* = 0.3,*β* = 0.15 (a solid red line); *α* = 0.3,*β* = 0.25 (blue ∘).

**Figure 14:**
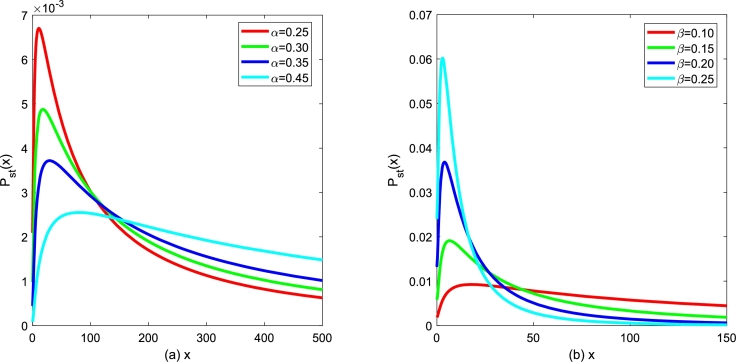
*P*_*st*_(*x*) versus *x* for various *α* and various *β*, respectively. Parameters are taken as *D*_2_ = 0.3,*Q* = 0.15,*λ*_2_ = 0.2,*τ* = 0.1 with (a) *β* = 0.1 and (b) *α* = 0.3.

Based on the program (27) - (29), the numerical solution of the improved Gompertz tumor growth system with two types of noise can be obtained. Next, SPD of the system will be further simulated and discussed. Fig. 12(a) and Fig. 12(b) depict SPD, $P_{st}(x)$, as a function of *x* governed by Eq. (5), and its analytic expression of SPD (10) are depicted by solid lines, while its numerical simulations are depicted by ∘. Fig. 13(a) and Fig. 13(b) show SPD, $P_{st}(x)$ governed by (14), and its analytic expression of SPD (19) is depicted by solid lines, while its numerical results are depicted by ∘. One can see that $P_{st}(x)$ starts to increase and then decreases gradually with *x* increasing, which indicates that the probability $P_{st}(x)$ of the tumor cell population is very high if the number of tumor cells *x* is small. However, with *x* increasing, the cell growth rate decreases due to the influence of tumor cell growth environment and other factors, and SPD, $P_{st}(x)$, gradually decreases and finally falls to zero. In Fig. 12 and Fig. 13 one can find that the analytical expression is consistent with the numerical simulation results. In addition, the influences of birth rate *α*, death rate *β*, noise intensity $D_{2},Q$, correlation coefficient $\lambda_{2}$ between two types of noise and correlation time *τ* on steady-state were discussed through Figure 14, Figure 16.

Furthermore, behaviors of analytic expression of SPD (19) will be discussed by influence of other parameters in Figure 14, Figure 16. In Fig. 14, the characteristics of SPD (19) for various *α* and *β* are depicted, respectively. The SPD curve displays non-monotonic behavior and reaches its maximum value at a certain *x* value. In Fig. 14(a), one can find that the peak value of SPD curve gets decreasing and flat, and gradually shifts to the right with the birth rate *α* increasing. The peak shape is relatively sharp, and its corresponding peak value of SPD is gradually higher if the *α* value is small. In Fig. 14(b), it is also found that the peak value SPD curve gradually moves to the left and becomes higher with the death rate *β* increasing. In conclusion, tumor cell growth and reproduction are heavily influenced by changes in *α* and *β*. However, it is difficult to control the natural birth and death parameters manually because they are affected by the micro-environment factors of individual survival. Thus, if one of these rates is known or can be estimated in advance, we can also consider looking for time-dependent functions that affect growth and mortality, representing the effect of treatment, indirectly altering natural birth and death rates.

**Figure 15:**
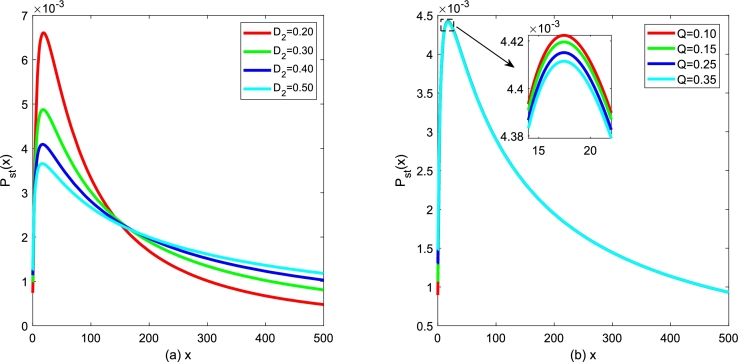
*P*_*st*_(*x*) versus *x* for various *D*_2_ and various *Q*, respectively. Parameters are taken as *α* = 0.3,*β* = 0.1,*λ*_2_ = 0.2,*τ* = 0.1 with (a) *Q* = 0.15 and (b) *D*_2_ = 0.35.

In Fig. 15, the characteristics of SPD (19) for various $D_{2}$ and *Q* are depicted, respectively. The SPD curve still displays non-monotonic behavior and reaches its maximum at some point. In Fig. 15(a), one can find that, with the external noise intensity $D_{2}$ increasing, the peak value SPD curve gradually shifts to the left and becomes lower, which indicates that the stability of the system weakens. In Fig. 15(b), one can also find that the size and position of the extreme value of SPD only produce a slight deviation with internal noise intensity *Q* increasing. This phenomenon indicates that SPD is much more sensitive to external noise intensity $D_{2}$.

Generally speaking, additive Gaussian white noise comes from the internal biological system and is the potential randomness of tumor cell growth in vivo. The multiplicative Gaussian colored noise comes from the external treatment and other environmental interference factors, among which the intensity of gaussian colored noise $D_{2}$ reflects the effect intensity of external treatment and immune cells on inhibiting the growth and diffusion of tumor cells. Therefore, the effects of random growth of tumor cells, external therapy, drug feedback, and other factors on tumor cell growth and diffusion were analyzed based on the kinetic equation, which has a particular guiding role in effectively inhibiting tumor cell proliferation.

**Figure 16:**
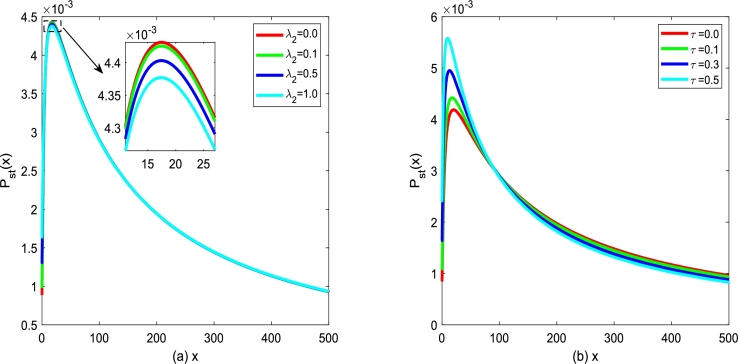
*P*_*st*_(*x*) versus *x* for various *λ*_2_ and various *τ*, respectively. Parameters are taken as *α* = 0.3,*β* = 0.1,*D*_2_ = 0.35,*Q* = 0.15 with (a) *τ* = 0.1 and (b) *λ*_2_ = 0.2.

In Fig. 16, the characteristics of SPD (19) for various $\lambda_{2}$ and *τ* are depicted, respectively. The SPD curve still displays an asymmetric single-peak structure, and SPD can also reach its maximum at a certain *x* value. In Fig. 16(a), one can find that the position and size of the peak value of SPD curve only produce a slight change with the cross-correlation coefficient $\lambda_{2}$ increasing. In Fig. 16(b), one can also find that the extreme value of SPD increases gradually, and the position of the peak of SPD shifts toward x→0 with self-correlation time *τ* increasing. This phenomenon indicates that increasing the autocorrelation time *τ* is beneficial to enhance the stability of the system. In general, the coupling coefficient $\lambda_{2}$ between the two noises reflects the ability of tumor cells to adapt to appropriate external treatments, compete with normal tissues for nutrients, and escape from the surveillance of immune cells. The control method of the autocorrelation time *τ* needs to be further developed.

## 3 Impacts of coupled two types noise on MFPT, mean, variance and skewness

In order to address the effects of coupling two types of noise on the improved Gompertz tumor growth system, the stationary mean value 〈x〉, the mean first-passage time (MFPT), normalized variance $\sigma^{2}$ as well as normalized skewness $\sigma^{3}$ of the tumor cell population *x* will be introduced and discussed in this section.

### 3.1 MFPT

MFPT is one of the noteworthy feature indexes to characterize the transient characteristics of stochastic complex systems. This indicates the nature of transition of complex system from an unstable tumor cell state ($x_{u}=1$) to a stable tumor cell number state ($x_{s}=e^{\frac{\alpha }{\beta }}$) of improved Gompertz tumor growth system (14). MFPT of the tumor cell growth system has been investigated in many works of literature and will not be repeated here if the autocorrelation time is zero. This paper focuses only on the case of non-zero autocorrelation time.

In this work, we will examine the time, MFPT, which it takes for a complex system in going from an unstable state ($x_{u}$) to a stable state ($x_{s}$) without any external input periodic signal. The approximate expression of MFPT [52], [53] of the complex system could be obtained according to the Kramers transition probability,

(30) $$T\left(x_{u}\to x_{s}\right)=\int_{x_{u}}^{x_{s}}\frac{\text{d}x}{H(x)P_{st}\left(x\right)}\int_{0}^{x}P_{st}\left(y\right)\text{d}y,$$

here $T\left(x_{u}\to x_{s}\right)$, MFPT, represents the average time required for the number of tumor cells to transition from unstable state $x_{u}$ to stable state $x_{s}$, and H(x) and $P_{st}(x)$ are given by Eqs. (17) and (18), respectively. Due to the fact that direct calculation of Eq. (30) is difficult, and considering the fact that $D_{2}$ and *Q* are much smaller than the barrier $\Delta \Phi_{2}\left(x\right)=\Phi_{2}\left(x_{1}\right)-\Phi_{2}\left(x_{2}\right)$, a fastest descent method is adopted, and MFPT could be expressed as

(31) $$T\left(x_{u}\to x_{s}\right)\approx \frac{2\pi }{\sqrt{\left|V^{\prime \prime }\left(x_{u}\right)V^{\prime \prime }\left(x_{s}\right)\right|}}\times \exp\left[\Phi_{2}\left(x_{u}\right)-\Phi_{2}\left(x_{s}\right)\right],$$

in which V(x) and $\Phi_{2}(x)$ are given by Eqs. (4) and (21), respectively.

Taking $x_{u}=1$ as the unstable state and $x_{s}=e^{\frac{\alpha }{\beta }}$ as the stable state, substituting Eqs. (4) and (21) into Eq. (31), the expression of MFPT of improved Gompertz tumor growth system (14) could be obtained

(32) $$T\left(1\to e^{\frac{\alpha }{\beta }}\right)\approx \frac{2\pi }{\sqrt{\left|\beta^{2}-\alpha \beta \right|}}\times \exp\left[\Phi_{2}\left(1\right)-\Phi_{2}\left(e^{\frac{\alpha }{\beta }}\right)\right].$$

**Figure 17:**
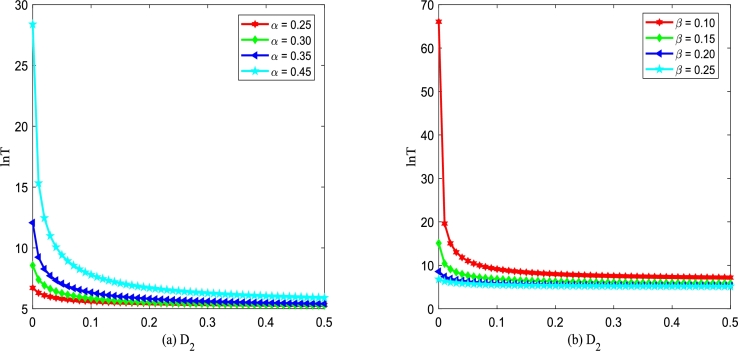
ln⁡T versus *D*_2_ for various *α* and *β*, respectively. Parameters are taken as *Q* = 0.15,*λ*_2_ = 0.1,*τ* = 0.2 with (a) *β* = 0.2 and (b) *α* = 0.3.

For convenient analysis, taking logarithm of Eq. (32), $\ln T(x_{u}\to x_{s})$ also describes MFPT. We draw the graph of the logarithm of MFPT with different noise parameters after numerical processing and discuss its influence on MFPT. The corresponding results are presented in Figure 17, Figure 20.

Figs. 17(a) and (b) show logarithm of MFPT, ln⁡T, a function of multiplicative noise intensity $D_{2}$ for various *α* and *β*. In Fig. 17, the logarithm of MFPT, ln⁡T, decreases rapidly and then achieves stability with multiplicative noise intensity $D_{2}$ increasing. From Fig. 17, ln⁡T is a monotone decreasing function of $D_{2}$, which indicates that increasing the multiplicative noise intensity $D_{2}$ is beneficial to the improved Gompertz tumor growth system (14) to implement transitions between two kinds of state. In addition, in Fig. 17(a), one can find that the smaller the birth rate *α* is, the better the improved Gompertz tumor growth system can achieve the transition between two states. In Fig. 17(b), one also can find that the larger the death rate *β* is, the more beneficial the improved Gompertz tumor growth system can achieve the transition between two kinds of state.

**Figure 18:**
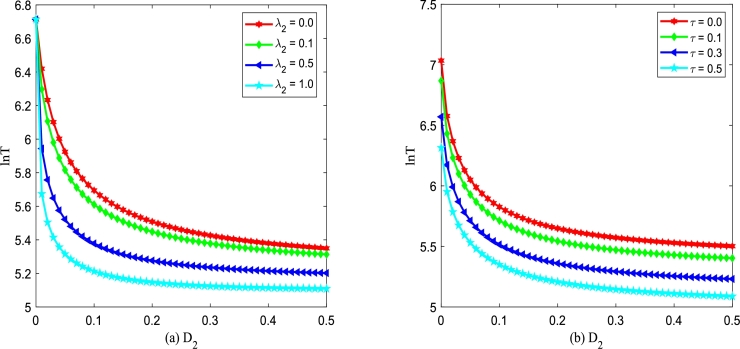
ln⁡T versus *D*_2_ for various *λ*_2_ and *τ*, respectively. Parameters are taken as *Q* = 0.15,*α* = 0.25,*β* = 0.2 with (a) *τ* = 0.2 and (b) *λ*_2_ = 0.1.

Figs. 18(a) and (b) show logarithm of MFPT, ln⁡T, versus multiplicative noise intensity $D_{2}$ with different $\lambda_{2}$ and *τ*. From Fig. 18, one can find that ln⁡T is a monotone decreasing function of $D_{2}$, which is similar to the results shown in Fig. 17. In Fig. 18(a), one can see that even if the Gaussian noise ξ(t) and η(t) are non-correlated, i.e., $\lambda_{2}=0$, ln⁡T declines and then achieves stability with $D_{2}$ increasing. In Fig. 18(a), significantly, one can also find that the greater the correlation $\lambda_{2}$ is, the more beneficial the improved Gompertz tumor growth system can achieve the transition from an unstable state to a stable state. Similarly, from Fig. 18(b) one can observe that the larger the correlation time *τ* is, the more favorable the improved Gompertz tumor growth system can achieve the transition from an unstable state to a stable state.

**Figure 19:**
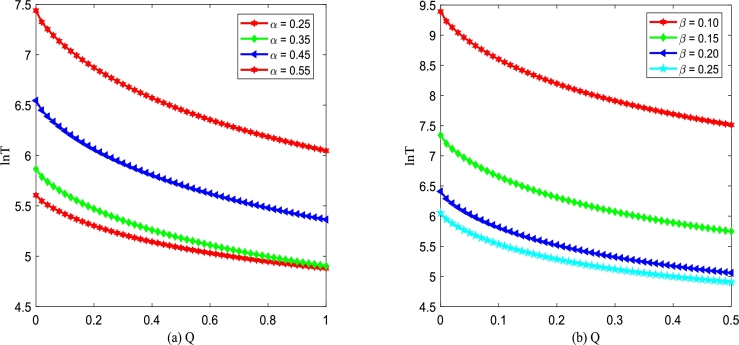
ln⁡T versus *Q* for various *α* and *β*, respectively. Parameters are taken as *D*_2_ = 0.35,*λ*_2_ = 0.1,*τ* = 0.2 with (a) *β* = 0.2 and (b) *α* = 0.3.

Figs. 19(a) and (b) demonstrate logarithm of MFPT, ln⁡T ($x_{u}\to x_{s}$) versus additive noise intensity *Q* with various *α* and *β*. Fig. 19 depicts that ln⁡T is a monotone decreasing function of *Q*. Significantly, from Fig. 19, one can find that increasing *Q* accelerates the phase transition of the improved Gompertz tumor growth system, shortening the mean time required for the transition of the number of tumor cells from unstable state $x_{u}$ to stable state $x_{s}$. This phenomenon indicates that additive noise plays an effective role in therapies. Additionally, from Fig. 19(a), it is found that the smaller the birth rate *α* is, the more beneficial the improved Gompertz tumor growth system can achieve the transition from unstable state $x_{u}$ to stable state $x_{s}$. In Fig. 19(b), one can also find that the larger the death rate *β* is, the more beneficial the system can achieve the transition between two kinds of states.

**Figure 20:**
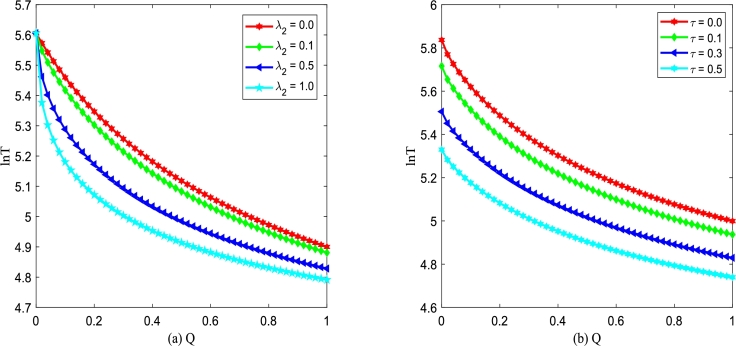
ln⁡T versus *Q* for various *λ*_2_ and *τ*, respectively. Parameters are taken as *D*_2_ = 0.35,*α* = 0.25,*β* = 0.2 with (a) *τ* = 0.2 and (b) *λ*_2_ = 0.1.

Figs. 20(a) and (b) demonstrate logarithm of MFPT, ln⁡T ($x_{u}\to x_{s}$) versus additive noise intensity *Q* with various $\lambda_{2}$ and *τ* changing. Fig. 20 shows ln⁡T is a monotone decreasing function of $D_{2}$, which is similar to the results shown in Fig. 19. In addition, in Fig. 20(a), one can find that the larger the correlation strength $\lambda_{2}$ between two types of noise is, the less time it takes for the improved Gompertz tumor growth system to achieve the transition from an unstable state $x_{u}$ to stable state $x_{s}$. From Fig. 20(b), one can also find that the larger the self-correlation time *τ* is, the more beneficial for the improved Gompertz tumor growth system can achieve the transition between two kinds of states.

### 3.2 The mean, variance and skewness

**Figure 21:**
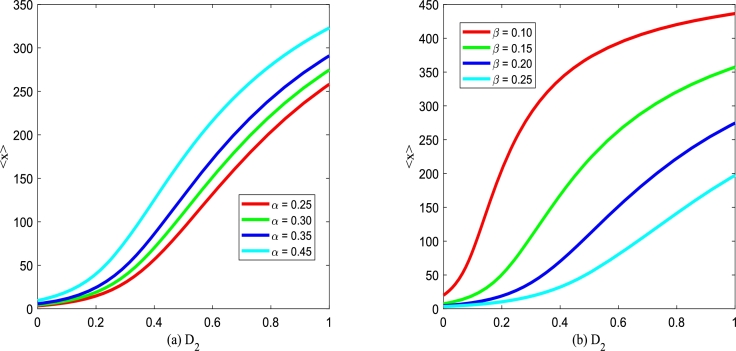
The mean 〈*x*〉 given by Eq. (33) versus *D*_2_ for various *α* and *β*, respectively. Parameters are taken as *Q* = 0.15,*λ*_2_ = 0.1, and *τ* = 0.2 with (a) *β* = 0.2 and (b) *α* = 0.3.

**Figure 22:**
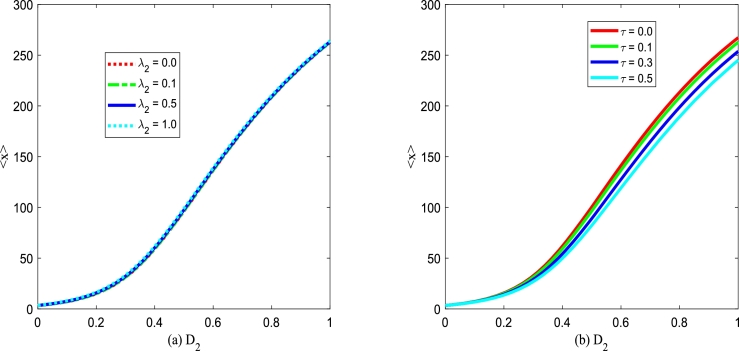
The mean 〈*x*〉 given by Eq. (33) versus *D*_2_ for various *λ*_2_ and *τ*, respectively. Parameters are taken as *α* = 0.25,*β* = 0.2, and *Q* = 0.15 with (a) *τ* = 0.2 and (b) *λ*_2_ = 0.1.

The change in the tumor cell can directly reflect the therapeutic effect, thus, it is essential to analyze the effect of noise on the number of tumor cells. To quantitatively address the influence of white Gaussian noise and colored noise on the number of tumor cells *x* governed by Eq. (14), we introduce the mean value of tumor cells through SPD function [54], [55], which can be expressed as follows

(33) $$\langle x\rangle =\int_{0}^{+\infty }xP_{st}(x)\text{d}x,$$

where $P_{st}(x)$ is defined by (18), and 〈x〉 denotes the average value of tumor cells. Increasing 〈x〉 indicates the continuous growth of tumor cells in immune monitoring, whereas the tumor continues to decay and die. In Figure 21, Figure 22, the average tumor cells 〈x〉 of is depicted versus Gaussian colored noise intensity $D_{2}$ with various birth rate *α*, death rate *β*, noise correlation intensity $\lambda_{2}$ and noise correlation time *τ*.

Figs. 21(a) and (b) plot the influence of the birth rate *α* and the death rate *β* on average tumor cells 〈x〉 versus multiplicative noise intensity $D_{2}$. In Figs. 21(a) and (b), one can find that the steady-state average tumor cells 〈x〉 increases gradually with multiplicative noise intensity $D_{2}$ increasing. In addition, Fig. 21(a) shows that increasing birth rate *α* results in the steady-state mean 〈x〉 of the tumor population gradually increasing, which indicates that increasing the size of birth rate *α* will promote the growth of tumor cells. Fig. 21(b) depicts that increasing *β* results in a gradual decrease in the steady-state mean 〈x〉 of the tumor population, which indicates that increasing the size of the death rate *β* inhibits tumor cell growth.

Figs. 22(a) and (b) plot the influence of cross-correlation coefficient $\lambda_{2}$ and self-correlation time *τ* between noises on average tumor cells 〈x〉 versus multiplicative noise intensity $D_{2}$. From Figs. 22(a) and (b), one can find that the steady-state average tumor cells 〈x〉 increases gradually with multiplicative noise intensity $D_{2}$ increasing. Significantly, Figs. 22(a) and (b) also show that the average tumor cells 〈x〉 almost does not change with $\lambda_{2}$ and *τ* increasing, which indicates that the correlation strength between noise $\lambda_{2}$ and autocorrelation time *τ* have less effect on the growth of tumor cells than birth rate and death rate.

We know that the normalized variance [54], [55] can be used to measure the deviation of a set of the values of the tumor cells. And the normalized variance $\sigma^{2}$ of the tumor cells is defined by

(34) $$\sigma^{2}=\frac{\langle {\left(x-\langle x\rangle \right)}^{2}\rangle }{{\langle x\rangle }^{2}}=\frac{\langle x^{2}\rangle }{{\langle x\rangle }^{2}}-1,$$

where the number of tumor cells *x* is governed by Eq. (14), and $\langle x^{2}\rangle =\int_{0}^{+\infty }x^{2}P_{st}(x)\text{d}x$.

**Figure 23:**
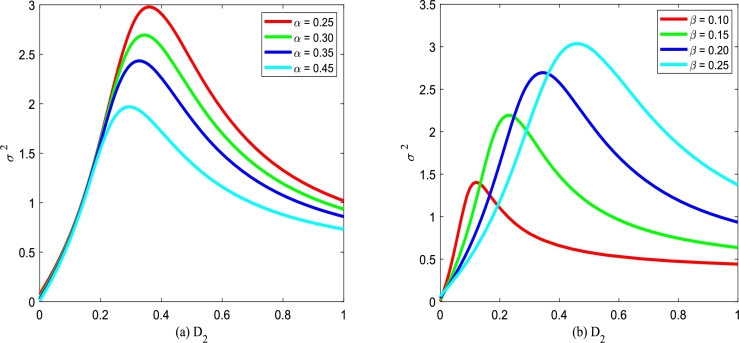
The normalized variance *σ*^2^ with Eq. (34) versus *D*_2_ for various *α* and *β*, respectively. Parameters are taken as *Q* = 0.15,*λ*_2_ = 0.1, and *τ* = 0.2 with (a) *β* = 0.2 and (b) *α* = 0.3.

The normalized variance $\sigma^{2}$ measures the deviation of a set of the values of the tumor cells. Fig. 23 plots that $\sigma^{2}$ of the tumor cells versus multiplicative noise intensity $D_{2}$ for various birth rate *α* and different death rate *β*. From Fig. 23, one can see that the curve of $\sigma^{2}$ versus $D_{2}$ exists a maximum value. In Fig. 23, one can find that $\sigma^{2}$ could generate large deviations by adjusting the birth rate *α* and the death rate *β*. Fig. 23(a) shows that the peak $\sigma^{2}$ declines with the birth rate *α* increasing, while Fig. 23(b) shows the peak $\sigma^{2}$ increases with the death rate *β* increasing. In a word, the smaller the birth rate *α* is, the larger normalized variance $\sigma^{2}$ is. However, the larger the death rate *β* is, the larger normalized variance $\sigma^{2}$ is.

**Figure 24:**
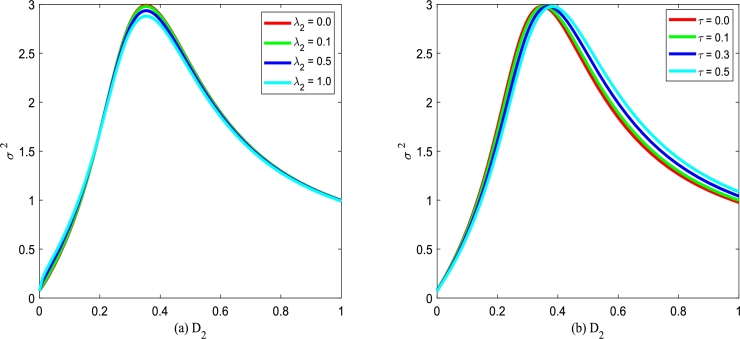
The normalized variance *σ*^2^ with Eq. (34) versus *D*_2_ for various *λ*_2_ and *τ*, respectively. Parameters are taken as *α* = 0.25,*β* = 0.2, and *Q* = 0.15 with (a) *τ* = 0.2 and (b) *λ*_2_ = 0.1.

Figs. 24(a) and (b) plot the normalized variance $\sigma^{2}$ versus multiplicative noise intensity $D_{2}$ with various cross-correlation coefficients $\lambda_{2}$ and various self-correlation time *τ*. From Fig. 24, one can also see that the curve of $\sigma^{2}$ versus $D_{2}$ exists a maximum value. However, $\sigma^{2}$ almost does not vary by adjusting the cross-correlation coefficient $\lambda_{2}$ in Fig. 24(a) or self-correlation time *τ* in Fig. 24(b). This phenomenon shows that the cross-correlation coefficient $\lambda_{2}$ and self-correlation time *τ* has little effect on the normalized variance $\sigma^{2}$, if system parameters and noise intensity are fixed.

It is well known that the normalized skewness [54], [55] is used to measure the asymmetry of the distribution of the tumor cells. And the normalized skewness $\sigma^{3}$ of the tumor cells is defined by

(35) $$\sigma^{3}=\frac{\langle x^{3}\rangle }{{\langle x\rangle }^{3}}-3\sigma^{2}-1,$$

where the number of tumor cells *x* is governed by Eq. (14), $\sigma^{2}$ is defined by Eq. (34), and $\langle x^{3}\rangle =\int_{0}^{+\infty }x^{3}P_{st}(x)\text{d}x$.

**Figure 25:**
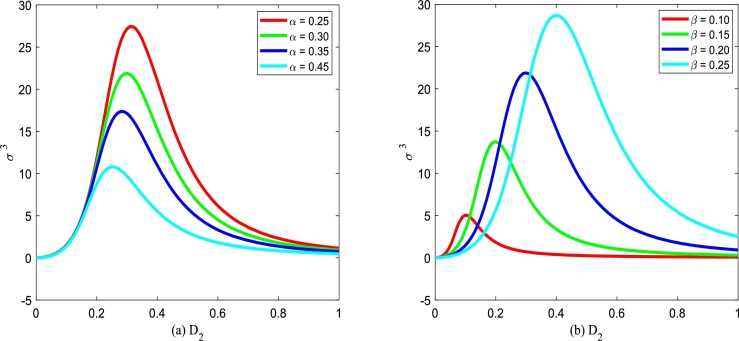
The normalized skewness *σ*^3^ with Eq. (35) versus *D*_2_ for various *α* and *β*, respectively. Parameters are taken as *Q* = 0.15,*λ*_2_ = 0.1, and *τ* = 0.2 with (a) *β* = 0.2 and (b) *α* = 0.3.

Fig. 25 plots $\sigma^{3}$ versus multiplicative noise intensity $D_{2}$ for various the birth rate *α* and various death rate *β*. In Fig. 25, one can see that the curve of $\sigma^{3}$ versus $D_{2}$ exists a maximum value. Fig. 25(a) plots the peak of $\sigma^{3}$ decreases with birth rate *α* increasing. Fig. 25(b) plots the peak of $\sigma^{3}$ increases and shifts toward right with the death rate *β* increasing.

**Figure 26:**
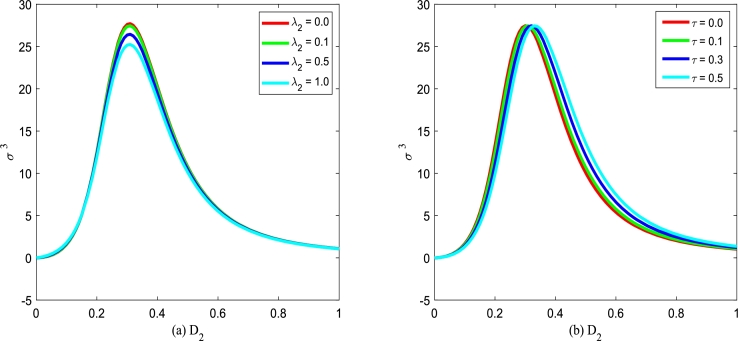
The normalized skewness *σ*^3^ with Eq. (35) versus *D*_2_ for various *λ*_2_ and *τ*, respectively. Parameters are taken as *α* = 0.25,*β* = 0.2, and *Q* = 0.15 with (a) *τ* = 0.2 and (b) *λ*_2_ = 0.1.

Figs. 26(a) and (b) plot the normalized skewness $\sigma^{3}$ versus multiplicative noise intensity $D_{2}$ with various correlation coefficients $\lambda_{2}$ and various correlation time *τ*. In Fig. 26, one can also see that there is a maximum value in the curve of $\sigma^{3}$ versus $D_{2}$. However, $\sigma^{3}$ almost does not change by adjusting cross-correlation coefficient $\lambda_{2}$ or self-correlation time *τ*. This phenomenon shows that cross-correlation coefficient $\lambda_{2}$ and self-correlation time *τ* have little effect on the normalization skewness $\sigma^{3}$.

## 4 Discussion and conclusion

Taking into account the fluctuations of certain factors in the growth process of tumor cells, in this work, we researched a stochastic improved Gompertz growth model. The expressions of SPD of an improved Gompertz tumor growth model with coupled two types of noise are obtained by the unified color noise approximation theory and FPE. Furthermore, numerical results of the improved Gompertz tumor growth model are fully discussed. Finally, the influences of coupled two types of noise on the MFPT, mean, variance, as well as skewness are also further investigated. It is noteworthy that the transient and steady-state properties of the above research are unique to the stochastic model, and do not have these properties in the deterministic model.

The main results and findings of this work are summarized below. Although tumor cell growth and reproduction are a very complicated process, this study demonstrates that the changes in system parameters and the addition of noise have a significant impact on the dynamic improved Gompertz tumor growth system. Our findings indicate that tumor cell proliferation may be successfully inhibited by a low birth rate *α* and a high death rate *β*. However, since the birth and death parameters of the system are affected by the micro-environmental factors of individual survival, and because the size of *α* and *β* varies with the individual body system, thus, it is not easy to control the size manually. But we can seek time-dependent functions that affect growth or death rate to estimate the effect of therapy if one of the rates is known or can be estimated previously. The results also have demonstrated that under the weak coupling coefficient, with the lowest utilization rate of surrounding micro-environmental factors in tumor growth, tumor cell growth can be inhibited. It is worthy to point out that tumor cells can maximize the utilization of surrounding micro-environmental factors and promote tumor growth with coupling coefficient increases. In addition, appropriate reduction of noise intensity can reduce the amount of tumor cell groups. Due to the fact that multiplicative noise is an external noise, thus, the form and intensity of noise can be artificially selected and controlled.

Specifically, during the growth of tumor cells, if patients can maintain a positive mood and good psychological state, they can enhance the immune function of the body through the regulation of the central nervous system, improve the anti-tumor resistant ability of the body, and promote the rehabilitation of tumor patients. Namely, this method controls the intensity of additive noise by changing patients' immunity. The intensity of the multiplicative noise can be controlled by increasing the dose of anti-cancer drugs, prolonging the time of action of drugs, and increasing the intensity of radiation in radiation therapy. In addition, competition for nutrients between normal and newly deteriorated tissues becomes intense during tumor tissue growth, so the coupling coefficient between the two noises can be adjusted by altering the nutrient supply of normal tissue and tumor cells. However, specific control methods of multiplicative noise intensity need further development. In conclusion, this study provides a theoretical basis for discovering therapeutic ways for tumor diseases.

It is remarkable to point out that the stochastic improved Gompertz tumor growth model considered in this paper only considers the natural growth rate *α* and *β* changes. However, the stochastic improved Gompertz tumor growth model including the effect of anti-tumor treatment is more valuable for research. The research is now underway. Understanding the dynamic growth characteristics of tumor cells can not only provide the theoretical basis for the growth and inhibition of tumor cells in clinical practice but also provide theoretical knowledge for the detection and treatment of tumor in clinical medicine and have certain guiding significance for the control and treatment of tumor diseases. In addition, it should also be pointed out that the steady-state analysis approach employed in this paper is general and applicable to many other types of single tumor models. However, a model with two or more types of tumor cell growth needs further exploration.

## Declarations

### Author contribution statement

Huijun Lv, Guitian He: Conceived and designed the experiments; Performed the experiments; Analyzed and interpreted the data; Contributed reagents, materials, analysis tools or data; Wrote the paper.

Hui Cheng, Yun Peng: Performed the experiments; Analyzed and interpreted the data; Contributed reagents, materials, analysis tools or data; Wrote the paper.

### Funding statement

Prof. Guitian He was supported by Sichuan Youth Science Project No. 2022NSFSC1840. Prof. Guitian He was supported by Natural Science Foundation of Guangxi Minzu University No. 2019KJQD02. Prof. Guitian He was supported by and Xiangsi Lake Young Scholars Innovation Team of Guangxi Minzu University No. 2021RSCXSHQN05. Prof. Guitian He was supported by 10.13039/501100001809National Natural Science Foundation of China 11601450, 11961006, 11526172. Prof. Guitian He was supported by 10.13039/501100004607Natural Science Foundation of Guangxi Province 2020GXNSFAA159100, AD21159013, 2021GXNSFAA220033.

### Data availability statement

No data was used for the research described in the article.

### Declaration of interests statement

The authors declare no conflict of interest.

### Additional information

No additional information is available for this paper.
